# Antiretroviral Therapy and Dyslipidaemia: Unlocking the Code

**DOI:** 10.1371/journal.pmed.0030085

**Published:** 2006-01-24

**Authors:** Patrick W. G Mallon

## Abstract

Mallon discusses a new study that describes associations between apoC-III polymorphisms and triglyceride concentrations in a large population of HIV-infected patients from a variety of ethnic backgrounds.

Dyslipidaemia, characterised by elevated total cholesterol, elevated non–high density lipoprotein (HDL) cholesterol, low HDL cholesterol, and elevated triglycerides, is common in patients with HIV who are treated with long-term antiretroviral therapy (ART) [ 1]. Although multiple factors influence lipid levels in patients treated with ART, exposure to protease inhibitors (PI) is thought to play an important role in the development of dyslipidaemia [ 2, 3]. Data showing a relationship between length of exposure to ART and increased incidence of cardiovascular events [ 4] have heightened concern that ART-associated dyslipidaemia will result in increased rates of cardiovascular disease among ART-treated patients with HIV.

The pathogenesis underlying ART-associated dyslipidaemia is thought to be related to drug-induced effects at the subcellular or molecular level [ 5–7]. This pathogenic mechanism, together with the interindividual variability in the prevalence and severity of ART-associated dyslipidaemia, suggests an important role for genetic factors in the pathogenesis of this dyslipidaemia. Small variations in genetic sequence, or single-nucleotide polymorphisms (SNPs), are common in the human genome [ 8]; many adverse drug reactions occur in response to drugs that are metabolised by enzymes known to contain functionally relevant polymorphisms [ 9]. Determining the effect of SNPs or groups of SNPs (halotypes) on an individual's response to either drugs or disease may help to limit these adverse drug reactions. This area has become a focal point of translational molecular research.

## Triglycerides and ApoC-III

Apolipoproteins (apo) are important components of circulating lipoproteins. ApoC-III, a 79–amino acid glycoprotein synthesised in the liver and small intestine, is a major constituent of chylomicrons and very low density lipoproteins (VLDLs), both of which are triglyceride-rich lipoproteins. ApoC-III regulates the synthesis and catabolism of VLDL, an important contributor to plasma triglyceride concentrations [ 10]. In human studies, levels of apoC-III showed a positive correlation with plasma triglycerides, and higher apoC-III concentrations have been associated with recurrent cardiovascular events [ 11]. In studies of people with HIV, apoC-III levels showed a positive correlation with triglyceride concentrations in males with HIV [ 12], and apoC-III levels in males treated with PI were two to three times higher than controls [ 13].

## Influence of Polymorphisms on ApoC-III Function

Regulation of apoC-III occurs at the level of its transcription. Insulin interacts with an insulin-responsive element in the promoter region of apoC-III, resulting in down regulation of apoC-III expression [ 10]. Within the insulin-responsive element, the presence of two apoC-III polymorphisms, -455T/C and -482C/T, affects the ability of insulin to down-regulate apoC-III expression in vitro [ 14]. These polymorphisms are common in populations infected with HIV [ 15, 16]. Along with another polymorphism in the 3′ untranslated region termed SstI (3238C/G), these polymorphisms have been associated with hypertriglyceridaemia in studies of both HIV-positive and -negative populations [ 10, 14–17].

Two previous studies in patients with HIV explored the relevance of apoC-III polymorphisms to ART-associated dyslipidaemia. In one study, 60% of individuals expressing all three apoC-III variants together with an apoE variant experienced extreme hypertriglyceridaemia (greater than 7 mmol/l) when exposed to ritonavir, values more than two times those of patients with similar genetic profiles not on ART treatment [ 16]. In another study, patients receiving PI who carried the -482T or -455C alleles had higher triglycerides (50%–60% higher) than patients with wild-type apoC-III [ 15].

Both studies had limitations. In the smaller study, the 60 patients were all White men prescribed PI-containing ART [ 15], and patients with pre-therapy dyslipidaemia were excluded. Only 21% of the data points studied were in patients taking ritonavir, a PI associated with dyslipidaemia [ 3] that is now increasingly used in small doses to pharmacologically “boost” the levels of other PIs. Although the larger study ( *n* = 329) included women (21%), the cohorts were still predominantly White (88%) [ 16]. As there are large racial/ethnic differences in the prevalence of not only HIV [ 18] but also metabolic syndrome and dyslipidaemia [ 19], it is also important to determine the different effects of potentially functional polymorphisms across racial/ethnic groups if pharmacogenetic and genomic knowledge is to be applied to the treatment of diverse populations. These studies were not designed to address these issues.

## A New Study in a Racially/ Ethnically Diverse Population

In the current issue of *PLoS Medicine*, Foulkes et al. describe associations between apoC-III polymorphisms and triglyceride concentrations in a large ( *n* = 626) population of patients with HIV from a variety of racial/ethnic backgrounds [ 20]. A strength of this study was that it derived samples and collected data through the Adult AIDS Clinical Trial Group ( AACTG) A5128 Protocol, which allows for storage of DNA for pharmacogenetic and genomic research from participants enrolled in a range of AACTG clinical trials [ 21]. In contrast to other studies, 19.3% of the population was Black/ non-Hispanic and 17.9% was Hispanic, allowing for investigations into the effect of the apoC-III polymorphisms both within and between different racial/ethnic populations.

Foulkes et al. found wide variability in genotype frequency among different racial/ethnic groups with wild type less common among Black/non-Hispanics (10.8%) than among White/non-Hispanics (45.5%) and Hispanics (35.1%). The pattern and severity of dyslipidaemia also differed between groups. Although the Black/non-Hispanic group had lower triglycerides, lower non-HDL cholesterol, and higher HDL cholesterol levels than White/non-Hispanics and Hispanics, they experienced more pronounced increases in triglycerides in response to PI-containing ART.

In contrast to previous literature, presence of variant apoC-III alleles in the Hispanic group was associated with a seemingly protective effect when exposed to PIs, as patients carrying these variant alleles had lower triglyceride values in response to PIs than those carrying wild type. Although seemingly counterintuitive, similar protective effects of variant allele combinations have been previously described in people who are HIV-negative [ 17], even though an underlying mechanism has yet to be determined. These data point to specific differences between racial/ethnic groups, not only in the prevalence of apoC-III polymorphisms, but also in the influence of these polymorphisms on PI-related hypertriglyceridaemia.

This study does have some limitations. For example, women were underrepresented (11% compared to the 25% of North American people living with HIV who are women [ 18]); only 10% of patients on PI therapy were on ritonavir; and, as the authors acknowledge, the study still lacked the power to detect small interaction effects within different racial/ethnic groups. Nevertheless, this study demonstrates specific differences between racial/ethnic groups in both the occurrence of dyslipidaemia on ART and the influence of genetic factors on the prevalence of PI-related lipid abnormalities.

## Pharmacogenetics and Antiretroviral Therapy

The study by Foulkes et al. is among an increasing number of pharmacogenetic studies in HIV. Genetic variation has been linked to the metabolism of antiretroviral drugs [ 22–24], drug hypersensitivity [ 25], and components of HIV-associated lipodystrophy [ 26–29] ( Table 1), although several of these studies were small, enrolled predominantly non-Hispanic/White men, and studied polymorphisms that occur infrequently within populations. As a result, conflicting associations have been found [ 16, 26–29], and the clinical applicability of results is unclear.

**Table 1:**
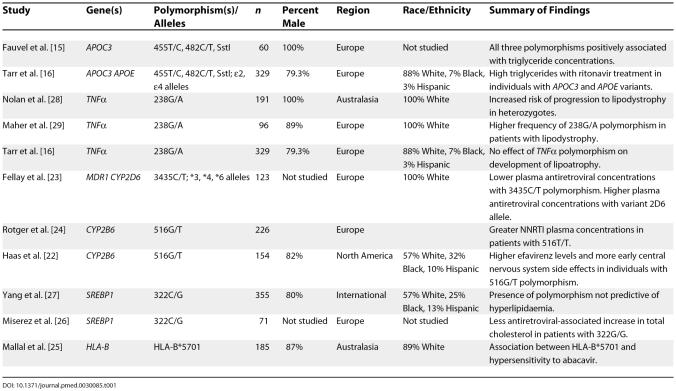
Pharmacogenetic/Genomic Studies in HIV

Notable exceptions include a study showing a high incidence of hypersensitivity to the nucleoside reverse transcriptase inhibitor abacavir in patients with the HLA-B*5701 halotype [ 25], and another study that demonstrated significant racial/ ethnic differences in the frequency of polymorphisms in the gene-encoding cytochrome P450 2B6, which is responsible for metabolism of the non-nucleoside reverse transcriptase inhibitor efavirenz. The differences in the frequency of these polymorphisms help explain the observed differences in metabolism of this drug between different racial/ethnic groups [ 22]. The latter study was also performed as part of the AACTG collaboration.

## Future Directions

Of the 43 million people estimated to be currently living with HIV, 43% are women, 64% live in sub-Saharan Africa, 18% live in South and Southeast Asia, and 4.5% live in Latin America [ 18]. Although we still have much to learn about the importance of race and ethnicity in the long-term treatment of HIV, studies such as that by Foulkes et al. underscore the importance of both race/ethnicity and genetic factors in the long-term treatment of HIV.

The study by Foulkes et al. underscores the importance of designing appropriately powered pharmacogenetic studies that include individuals from a wide variety of racial/ethnic backgrounds in order to determine the clinical importance of genetic variation. Although DNA banks raise ethical challenges [ 30], collaborations such as the AACTG are an ideal framework in which to perform these studies. Establishing and supporting similar collaborative groups in other regions of the world is essential if further useful studies are to be performed in this field. Such studies are vital if the eventual goal of safe long-term therapy for HIV is to be realised worldwide.

## Abbreviations

AACTG: Adult AIDS Clinical Trial Group
apo: apolipoprotein
ART: antiretroviral therapy
HDL: high density lipoprotein
PI: protease inhibitor
SNP: single-nucleotide polymorphism
VLDL: very low density lipoprotein
